# Sustainable pathway towards large scale melt processing of the new generation of renewable cellulose–polyamide composites

**DOI:** 10.1039/d0ra07141b

**Published:** 2020-12-24

**Authors:** Valentina Sessini, Bashar Haseeb, Antal Boldizar, Giada Lo Re

**Affiliations:** Department of Organic and Inorganic Chemistry, Faculty of Pharmacy, University of Alcalá 28805 Alcalá de Henares Madrid Spain; Department of Industrial and Materials Science, Chalmers University of Technology Rannvagen 2A SE-412 96 Gothenburg Sweden giadal@chalmers.se; Wallenberg Wood Science Center (WWSC), KTH Royal Institute of Technology Teknikringen 56 SE-100 44 Stockholm Sweden

## Abstract

Modern society's growing demands for accountable high-performance and more environmentally friendly materials is leading to increased interest and fast development of sustainable polymeric composite materials. New generations of “greener” products originating from renewable resources fulfil emerging requirements of low environmental and health & safety impacts and contribute to diminishing global dependence on fossil feedstock. The preparation of sustainable polymeric composites *via* reliable and reproducible melt-compounding methods is still challenging but has the potential to yield applicable and market competitive products. This literature survey reviews the current state of research involving the use of cellulosic materials, as bio-sourced and sustainable reinforcement in melt-processed polyamides and focuses on the main hurdles that prevent their successful large-scale melt-compounding. Particular emphasis is dedicated to emerging bio-sourced polyamides fitting the performance of engineering materials and at the same time offering additional interesting properties for advanced applications such as piezoelectricity for transducers, sensors, actuators and energy harvesters.

## Abstract 2

Biosourced polyamide/cellulose composites are promising engineering piezoelectric materials large-scale processable by sustainable, cost-efficient, and solvent-free wet-assisted melt-compounding.

## Graphical abstract

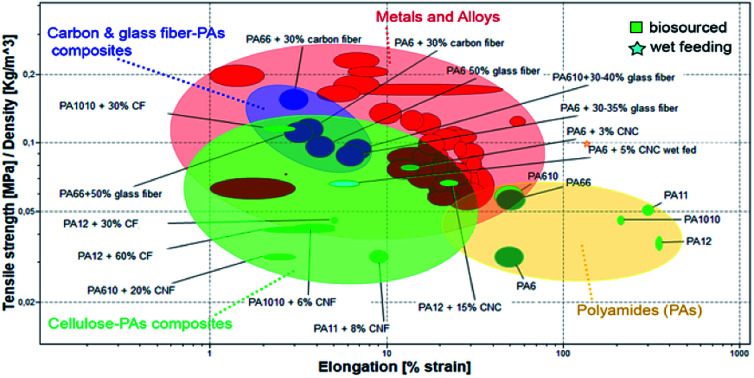

## Introduction

Research in the development of sustainable polymeric composite materials has received increased academic and industrial interest in recent decades. This is due to modern society's growing demands for accountable high-performance materials as well as more environmentally conscious consumers, industries and governments. Products, in general, are sought to be “greener” or in other words originate from renewable resources, have a low environmental impact and prompt low health & safety concerns. These requirements trail sustainability concerns as well as diminishing global supplies of fossil feedstock resources such as crude oil.^1,2^ A straightforward research approach to produce polymeric composite materials that fulfil these requirements is the use of feedstock from sustainable natural resources. The challenge in its essence being their processing *via* reliable and reproducible methods, yielding applicable and market competitive products. Currently, conventional melt processing techniques are assumed to continue being key processing methods for the large-scale production of thermoplastic composites. Extrusion and injection moulding are well established industrial-scale facilities considered sustainable because are inexpensive, fast, and organic solvent-free techniques.

Sustainability aiming thermoplastic composite products can regard cellulosic materials as ideal reinforcements.^3^ Being bio-sourced, they are abundant as well as biodegradable, and in many instances exhibit favourable mechanical properties when compared to many synthetic counterparts with the added benefit of a lower cost and light weight.^4,5^ Recent scientific advances in the production of cellulose nanomaterials such as cellulose nanocrystals (CNC) and cellulose nanofibrils (CNF) have shown numerous new possibilities for wide range of applications.^2,6,7^

Polyamides (PAs), commonly referred to as nylons, are of interest as polymer matrices due to their excellent mechanical and thermal properties as well as their relative ease of processing.^8–10^ Application wise, many polyamides and composites thereof are recognized as engineering grade materials which can in many instances even replace metal parts.

Extruded and moulded polyamide composites are currently found in a wide range of technical applications *e.g.* in automotive parts, electrical components, and food packaging. The increased availability of bio-sourced polyamides revamps them as viable sustainable materials today.^11^

**Fig. 1:**
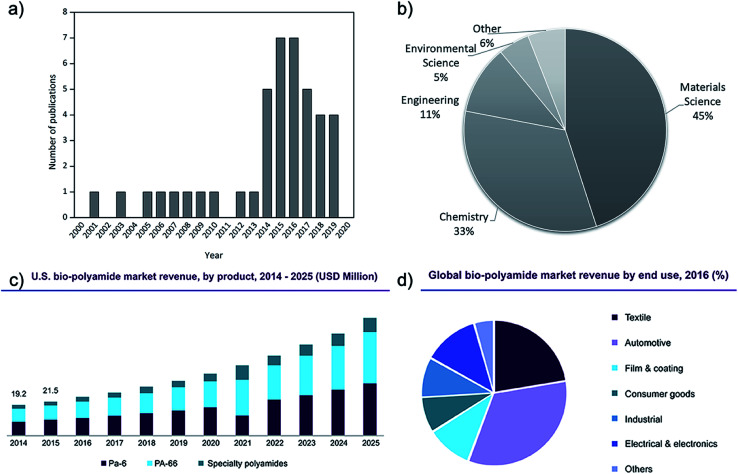
Number of relevant publications on melt processed polyamide and cellulose composites (Scopus database, 2000–today), (a) over the year of publication and (b) over the research field. (c) U.S. biosourced polyamide market profits by product (2014–2025). (d) Global biosourced polyamide market profit by end use in 2016.^13^

The research interest in cellulose–polyamide composites started in earnest in the 1980s.^12^ After a decline of interest mainly due to the temperature sensitivity of cellulose setting challenges for its melt processing with polyamide, research publications in this topic has intensified in the last five years (Fig. 1).

This survey on the current state-of-the-art on cellulose–polyamide composites focuses on the main hurdles that prevent their successful large-scale melt-compounding. Different research approaches and challenges are presented to support the development of a new generation of environmental-friendly materials bases on sustainable processing and feedstocks with advanced properties.

### Polyamides and biosourced polyamides

Polyamides are semi-crystalline thermoplastics characterized by a repeating polar amide group, (–CONH–). Since its first introduction in the market as a moulding material in 1940s, polyamides have been improved, being nowadays rather common engineering thermoplastics. Synthetic polyamides (*e.g.* PA6, PA66, PA46 and PA612) have been of interest as polymer matrices for composites with wide array of fillers and reinforcements due to their excellent mechanical and thermal properties, relative ease of melt processing (albeit at temperatures above 240 °C), relatively good adhesion to reinforcements, resistance to oils and corrosive chemicals, and attractive surface appearance.^8–10^ Polyamides and composites thereof have been used in a variety of packaging, engineering electrical, textile medical and auto applications including more demanding applications such as gas pipes and offshore oilfield. Properties and wide application range of high performance class nanofiller reinforced polyamides has been reviewed recently by Francisco *et al.*^14^ The most commonly traded grades of polyamides are PA6.6, PA6, PA66, Kevlar and other PAs such as the biosourced PA11, and their market is expected to register a compound annual growth rate (CAGR) of over 4% during the period 2016–2024.^15^

Biosourced polyamides (*e.g.* PA11, PA1010, PA410 and, to a lesser extent, PA610), as the name implies, are sourced from fully or partially derived renewable feedstock. These materials inherit the characteristic PAs properties, exhibiting high mechanical strength and thermal performance with added processing advantages consequently opening up new opportunities in their future market.^11,16^ The global biosourced PA market size was valued at USD 110 million in 2016 and it is expected to reach USD 220 million by 2022, according to a new study by Grand View Research, Inc. These values represent a predicted CAGR of 12.2% from 2015 to 2022, supporting the future increasing of their demand (Fig. 1c). The global production capacity of bio-PA is similar to that of the common bioplastics such as biobased PE, PLA and PBAT, being around 12% of the 2.11 million tonnes of bioplastics produced in 2019 (one percent of the more than 359 million tonnes of plastic produced annually).^13^ In general, biosourced PAs have somewhat lower melting temperatures, density, ductility, and moisture absorption than widely used synthetic nylons *e.g.* PA6 and PA66. Thanks to their renewability, recyclability, light weight, inexpensive nature, electromechanical resistivity, ductility, and creep resistance, biosourced PAs have been attracting attention from various end-use sectors and they are used in a wide variety of applications (Fig. 1d). Textiles emerged as the second-largest end-use segment in 2016, after automotive applications, while the electrical & electronics sector is projected to emerge as one of the fastest growing end-use sectors in the next few years.^13^

However, the main disadvantage to these biosourced materials is that they are currently more expensive compared to traditional nylons. The common biosourced PA11 is a castor oil-based biopolymer and is a semi crystalline polymer that exhibits six different crystalline phases.^17^ Its degree of crystallinity and phase composition can have a significant influence on its exhibited mechanical properties, giving it a wide selection of adjustable usages.^18^ In their study, Zhang *et al.*^19^ showed that there is an optimum annealing temperature for PA11, around 165 °C, when crystallinity can be maximized. Although PA11 represents only a minor portion of global nylon production, the demand for PA11 and biosourced polyamides is expected to grow (Fig. 1c), making them of interest for research within both academia and industry. In fact, the rises in oil price and the increasing environmental awareness as well as the stricter environmental policies will make fossil-based polymers more expensive, favouring the production of bio-based alternatives. The production of traditional PA has higher environmental impact than those of biobased PA contributing the potential global worming (≈7 Kg CO_2_ eq. per kg PA 12 against ≈4 Kg CO_2_ eq. per kg bio-PA 12).^11^

### Cellulose as reinforcement

Cellulose is a natural biopolymer and is a main constituent of plant cell walls, tunicates, and many species of bacteria where it serves as the fundamental structural reinforcement. The combined global annual production of cellulose by plants is estimated to be 1.5 × 10^12^ tons, making cellulose the most abundant polymer on earth.^3^ It can therefore be regarded as a rich source of materials for numerous applications and as such is currently receiving significant attention in the context of sustainability.^20,21^ Cellulose and its numerous derivatives have been extensively studied, focusing on their biological, chemical, as well as mechanical properties.^22–24^

**Fig. 2:**
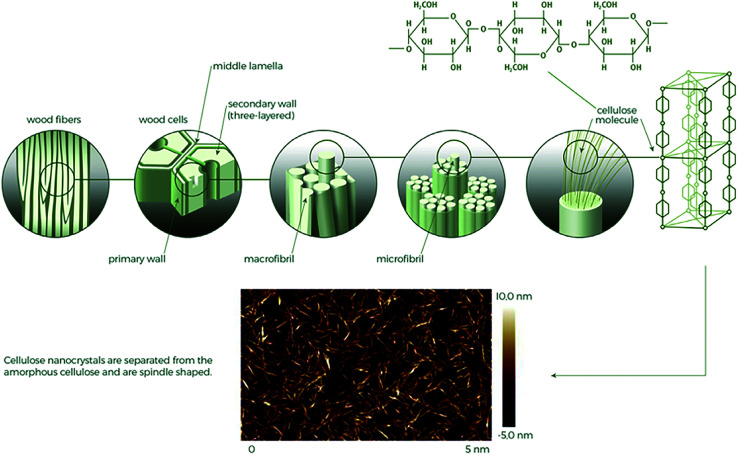
Different levels of the cellulose structure in a typical wood source including an enhanced AFM image of liberated cellulose nanocrystals from a wood source.^26,27^

Cellulose polymeric molecule is recognized as a homo-polysaccharide composed of d-anhydroglucopyranose repeating units linked by glycosidic bonds, produced *via* condensation polymerization of glucose.^3,22,25^ Its linearity and many hydroxyl groups enable the formation of ordered crystalline structures which provide the unique mechanical properties to cellulose fibres (CF). Different levels of the hierarchical structure in cellulose fibres found in a typical plant source is shown in Fig. 2.

The polymers combine to form long and continuous hydrogen bond stabilized microfibrils, and in turn several microfibrils self-assemble in macrofibrils, which can be found oriented in varying directions.^28^ Combinations of three main polymers compose the different layers of the plant cell wall: cellulose, hemicellulose, and lignin. The cells are bound together by a middle lamella consisting of the polysaccharide pectin.^2,25^

Pulp and its various forms^29,30^*e.g.* thermomechanical pulp and Kraft pulp are generally derived by the mechanical and/or chemical separation of individual CF found in cellulose rich sources such as wood or cotton. The chosen production methodology yields fibres with different compositions (*e.g.* cellulose, hemicellulose and lignin ratios), surface chemistries and physical properties. In the mechanical pulping process, temperature, humidity and mechanical forces are used to soften the fibres followed by their separation by shearing force with minimal loss of fibres. In the chemical pulping process, caustic alkaline chemicals are used to solubilize the lignin and most carbohydrates in the middle lamella which hold the fibres together, resulting in a lower pulp yield but in general liberating stiffer fibres. These different features affect their thermal stability and dispersibility in a polymer melt, and in turn their reinforcement effect.

Further chemical and/or mechanical deconstruction at the microfibril level results in nanocelluloses which are defined as cellulosic materials that possess at least one dimension in the nanometric scale such as “microfibrillated cellulose” (MFC) (also referred as microcrystalline cellulose, MCC), cellulose nanofibrils (CNFs), and cellulose nanocrystals (CNCs).^3,23,31^

**Fig. 3:**
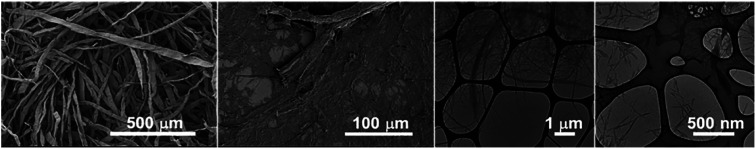
Pristine morphologies of the cellulosic materials used in this study. In particular: (a) bleached pulp (bar 500 microns), (b) microfibrillated cellulose (bar 100 microns), (c) enzymatic nanofibrils (bar 1 micron), and (d) cellulose nanocrystals (bar 500 nanometres). This figure has been adapted from ref. 32 with permission from American Chemical Society, copyright 2018.

Pristine morphologies of the different cellulosic materials are showed in Fig. 3, and their main properties reported in Table 1.

**Table 1:** Properties of wood-based materials: PULP,^29,33^ MFC,^34–36^ CNFs and CNCs^37,38^

Property	PULP	MFC	CNFs	CNCs
Diameter	15–50 μm	2.5 nm to 25 μm	3–60 nm	2–5 nm
Length	>2000 μm	0.1–30 μm	1–15 μm	10–250 nm in plant 100–10 000 nm (tunicate-algae-bacteria)
Crystallinity [%]	45–70	35–55	<50	60–90
Thermal stability [°C]	200–270	200–250	Up to 260	Up to 230
Tensile modulus [GPa]	32–40	8–14	23	∼150
Tensile strength [GPa]	0.08–0.13	0.12–0.24	0.3–0.4	7.5–7.7
Density [g cm^−3^]	1.2	1.2–1.4	1.4–1.5	1.5–1.6

MFC are characterized by a non-homogeneous size distribution, and only some of the microfibrillated fibrils are on the nanoscale level. As a consequence, the MFC average aspect ratio is in the range of 1 to 2, limiting their reinforcement effect in composites, while preserving some of the features of the nanocellulose, *i.e.* early gelation in water dispersion and hornification upon drying (as described further below).^32^

CNCs and CNFs are typically distinguishable by the way they are obtained and resultant amorphous material content. CNFs are obtained by mechanical disintegration, or defibrillation, often facilitated by chemical or enzymatic pre-treatment and they are characterized by a residual intra-crystallin amorphous content.^20,25,37^ CNCs are typically obtained by acid hydrolysis treatment leading to the elimination of most of the amorphous regions and leaving spindle shaped structures.

The properties of the nanocellulose are strongly determined by their surface features which depend mainly on the raw material used and its preparation process. For example, it is worth noting that hydrophobic compounds are usually still present on the surface of CNFs and that a surface charge may be fixed by a pre-treatment step. Another example is that sulfuric acid is classically used for CNC production because it promotes the formation of negatively charged sulphate groups at the surface of the released crystals, resulting in very stable aqueous dispersions. However, hydrolysis with sulfuric acid causes the introduction of a considerable amount of negatively charged sulphate half-ester groups on the CNC surfaces which catalyse the thermal degradation during melt processing, especially for polyesters.^39^ Hydrolysis of CNCs with hydrochloric or phosphoric acid instead may introduce phosphorylated CNCs with a much lower surface charge density and higher thermal stability.^39–41^ Other strong acids have been used to produce CNCs with other surface moieties that exhibit different chemical qualities, such as hydrobromic and phosphotungstic or other organic acids.^22,24,39^

Cellulose nanomaterials are often obtained as very dilute suspensions (typically <2 wt%), usually in water because it is a convenient, no toxic and inexpensive polar liquid medium. If the concentration increases with only a few percent solid content, the viscosity of the dispersion increases sharply resulting in gelling. Upon drying, the nanoparticles aggregate through a to substantial and irreversible fibre and fibril hydrogen bonding (also called hornification,^42,43^ see section below), and the nanoscale is irreversibly lost. Freeze-drying, and other similar methods, have been used to circumvent the aggregation of nanomaterials to preserve their individualized state.^44,45^

The use of nanocellulose materials as biosourced green reinforcements in plastic composites has garnered particularly significant research interest during the last 10 years.^2,7,16,31,37,46–51^ The intrinsic benefits of nanocellulose which are often mentioned in the literature include, such as type variability, low density, very high specific strength and modulus (rivalling that of steel^52^) and a high capacity for surface modification and energy generation by burning after usage.^21^ The size reduction in cellulosic nanomaterials retains and amplify most of these properties *e.g.* mechanical stiffness and the specific surface area, leading to a substantial increase of the available surface hydroxyl groups. This increased hydrophilic character further limits their adhesion and dispersion in non-polar matrices. Nanocelluloses are more thermally stable than wood fibers (WF) or flour because they are purified from most of the lignin, hemi-cellulose or other chemically and thermally liable constituents. However, their thermal stability is still low in relation to typical polymer melt processing temperatures, as for polyamides. Nanocelluloses are currently on their way to becoming a fully commercial and widely available products with a multitude of applications.^22^ An in-depth review of the large-scale production of nanocellulose and its economic aspects can be found in the review by de Assis *et al.*^20,25^

### Melt processing of cellulose reinforced polyamides

While several laboratory-scale methods have been used to produce polyamide-based composites reinforced with cellulosics having attractive mechanical properties, *e.g. via* solution casting,^53–55^ few of these processes can be considered industrially viable. The direct melt compounding of polymers and cellulose is in general considered more economical and formulation flexible. They involve established industrial-scale facilities with standard approaches such as extrusion and injection-moulding which are inexpensive, fast, and organic solvent-free techniques. Tables 2–4 include relevant studies on cellulose reinforced polyamides.

**Table 2:** Melt processing strategies and parameters of cellulose-reinforced polyamides

Author – year article	Matrix	Cellulose type	Processing aid/compatibilizer/surface functionalization	Processing techniques	Cellulose (wt%)
(Klason, Kubát, & Strömvall, 1984)^12^	PA6, PA12	Wood flour, bleached cellulose flour, bleached CF, all oven dried@105 °C/24 h	Neat	Internal mixer, extrusion (single-screw), injection moulding [PA12 max 195 °C; PA6 max 235 °C]	10.0–30.0
(Jacobson, Caulfield, Sears, Underwood, & Caufield, 2001)^70^	PA6, PA66	CF – oven dried at 105 °C	Processing aid: 0003–0005 wt% of carboxy methylcellulose (CMC) viscosity modifier	Extrusion (twin-screw), injection moulding [max 232 °C]	33.0
(Lu, Doyle, & Li, 2007)^58^	PA12	Wood flour, oven dried@103 °C/24 h	Neat	Internal mixer, compression moulding [max 200 °C]	40.0–60.0
(Xiaolin, 2008)^65^	PA6, PA66	CF – oven dried@120 °C/4 h	Neat binder: polyurethane aq. dispersion-Hydrosize U1-01, plasticizers: (NBBSA), caprolactam, thiurea, ceramic powder (INTEC SB 94), lithium chloride (LiCl)	Extrusion (twin-screw), injection moulding, compression moulding [max 270°]	10.0–30.0
(Chen, Gardner, 2008)^135^	PA6 & PA66 blend	Wood flour	Processing aid, 7 wt% (unknown)	Extrusion (single screw), (Davis standard Woodtruder) [max N/A]	43.0
(Arcaya *et al.*, 2009)^136^	PA6, PA66	Natural fibers (flax), pulp, CF, all vacuum oven dried (80 °C)	Neat	Internal mixer, injection moulding [max 265 °C]	20.0–35.0
(Panaitescu, Frone, & Nicolae, 2013)^57^	PA11	CNF – vacuum oven dried (60 °C) [H_2_SO_4_]	Neat	Internal mixer, two-roll mill, compression moulding [max 190 °C]	1.0–8.0
(Kiziltas, Gardner, Han, & Yang, 2014)^59^	PA6	MCC – oven dried@105 °C/16 h	Lubricant – fatty acid esters (5 wt%)	Internal mixer, injection moulding [max 250 °C]	2.5–30.0
(Bledzki, Feldmann, 2014)^137^	PA1010, PA 610	Man-made CF	Neat	Pultrusion, extrusion, (single screw), injection moulding [max 245 °C]	15.0–30.0
(Panaitescu, Gabor, Frone, & Vasile, 2015)^18^	PA11	CNF– vacuum oven dried (60 °C) [H_2_SO_4_]	Neat	Internal mixer, two-roll mill, compression moulding [max 190 °C], Annealing@165 °C/1 h	1.0–8.0
(Zhu, Kiziltas, Lee, & Mielewski, 2015)^16^	PA1010, PA 610	CNF – oven dried@70 °C	Neat [masterbatch, N/A wt%]	Extrusion (single screw), injection moulding [max 235 °C]	2.0–20.0
(Peng, Gardner, & Han, 2015)^56^	PA6	MCC – spray dried, CNF – spray dried, CNC – spray dried + oven dried@105 °C/12 h	PA processing aid-viscosity lowering agent (3 wt%)	Internal mixer, micro-injection moulding [max 270 °C]	2.5–10.0
(Nicharat, Sapkota, Weder, & Johan Foster, 2015)^41^	PA12	CNC – freeze dried [H_3_PO_4_ & H_2_SO_4_]	Neat	Internal mixer, compression moulding [max 190 °C]	5.0–20.0
(Armioun *et al.*, 2016)^78^	PA11	Wood fiber – oven dried@105 °C/2 h	Maleic anhydride grafted polypropylene (4 wt%)	Extrusion (twin-screw), injection moulding [max 210 °C]	10.0–30.0
(Yousefian & Rodrigue, 2016)^64^	PA6	CNC – oven dried@70 °C/24 h	Neat [masterbatch 10 wt%]	Extrusion (twin-screw), injection moulding [max 230 °C]	1.0–7.0
(Zierdt *et al.*, 2017)^138^	PA11	Wood fiber	Neat	Internal mixer, extrusion (twin-screw),Injection moulding [max 220 °C]	50.0
(Fernandes *et al.*, 2017)^139^	PA6	CF – bleached and non-bleached	Lubricant ethylene bis-stearamide (1 wt%)	Extrusion (twin-screw), injection moulding [max 250 °C]	20.0–40.0
(Yousefian *et al.*, 2017)^140^	PA6	CNC – oven dried@70 °C/24 h	Neat foaming agent: azodicarbonamide (1–2 wt%) [masterbatch 5 wt%]	Extrusion (twin-screw), injection moulding [max 230 °C]	0.5–5.0
(Rohner *et al.*, 2018)^67^	PA11	Wood CNF – freeze dried, spinex grass CNF – freeze dried	Neat	Extrusion (twin-screw), injection moulding, compression moulding [max 215 °C]	0.1–0.5
(Xu *et al.*, 2018)^141^	PA6	Wood fiber, oven dried@103 °C/24 h	Lithium chloride (LiCl) (0–3%), chain extender: 2,2′-(1,4-phenylene)bis(2-oxazoline) (PBO) (0.2–1%)	Internal mixer, compression moulding [max 235 °C]	20.0–40.0
(Beg *et al.*, 2018)^142^	PA6.10	MCC, oven dried	Impact modifier (IM), Exxelor VA1803 (2.0–5.0%)	Extrusion (twin-screw), injection moulding [max 240 °C]	20.0–40.0
(Oliver-Ortega *et al.*, 2018)^143^	PA11	Wood fiber	Neat	Kinetic mixer (twin-screw), injection moulding [max N/A]	20.0–60.0
(Gohn *et al.*, 2019)^81^	PA12	CNC	Neat	Internal mixer, compression moulding [max 200 °C]	5.0
(Venkatraman *et al.*, 2019)^144^	PA11	CNC	Neat	Planetary ball milling, internal mixer, compression moulding [max 200 °C]	10.0
(Annandarajah *et al.*, 2019)^145^	PA6	CF	Polypropylene maleic anhydride grafted polypropylene (PPgMA) [70 : 30 wt% ratio of PA6 : PP and 6 wt% PPgMA]	Extrusion (twin-screw), injection moulding [max 220 °C]	30.0
(Beg *et al.*, 2019)^146^	PA6.10	MCC, oven dried	Coupling agent: Exxelor VA1803 (5.0%), coupling agent: Bondyram 7103 (BR)	Extrusion (twin-screw), injection moulding, [max 240 °C]	30.0

**Table 3:** Summary of strategies and parameters of wet feeding approaches during melt processing of cellulose reinforced polyamides

Author – year article	Matrix	Cellulose type	Processing aid/compatibilizer/surface functionalization	Processing techniques	Cellulose (wt%)
(Winata *et al.*, 2003)^61^	PA6	CF –never dried (TerraCel™ 10J)	Neat + wollastonite	Extrusion (twin-screw), microcellular injection moulding (MuCell) [max 210 °C]	20.0–28.0
(Lee, Yoon, Lee, Lim, & Kim, 2014)^88^	PA6	CNF – never dried	Silane coupling agent: *N*-(β-aminoethyl)-γ-amino-propyltrimethoxysilane (Z-6020® Dow corning), 0.1; 0.5, and 1.0 wt% in water	High temperature-pressure calendaring [max 260 °C]	20.0–40.0
(Clemons, 2015)^60^	PA6	CNC – never dried, CNC – freeze dried	Neat	Micro internal mixer, micro injection moulding	5.0
(Peng, Walsh, Sabo, Turng, & Clemons, 2016)^69^	PA6	CNC – never dried	Neat	Extrusion (twin-screw), injection moulding, micro injection moulding (MuCell) [max 235 °C]	0.5–3.5

**Table 4:** Compatibilization strategies

Author – year article	Matrix	Cellulose type	Processing aid/compatibilizer/surface functionalization	Processing techniques	Cellulose (wt%)
(Paunikallio. 2006)^62^	PA12	CF (viscose)	Surface mod. – coupling agent: silyl coupling agent, aminosilane [(3-aminopropyl)triethoxysilane] then oven dried [in gas & liquid phase]	Micro-extruder (twin-screw), micro-injection moulding [max 215 °C]	40.0
(Li *et al.*, 2012)^147^	PA6	Wood flour (fir flour/SiO_2_ hybrid material) (FSHM)	Aminopropyltriethyoxysilane epoxy resin	Extrusion (twin-screw), injection moulding [max 235 °C]	3.0–25.0
(Corrêa *et al.*, 2014)^72^	PA6	CNC-freeze dried	Third component – macromolecule: coated with PA6 (*via* formic acid solution) [masterbatch 33 wt%]	Extrusion (twin-screw), injection moulding [max 260°]	1.0
(Semba *et al.* 2014)^66^	PA12	CNF-dried [temp N/A]	Surface mod. – grafting: reactive cationic quaternary ammonium salt, epoxy functionalized; 0.5%	Extrusion (twin-screw), injection moulding [max 190 °C]	5.0
(Leszczyńska. 2015)^63^	PA410	MFC	Surface mod. – acetylation: *via* acetic anhydride in toluene after multiple solvent exchanges – then freeze-dried	Micro-extruder (twin-screw) [max 250°]	1.0–5.0
(Feldmann, Heim, & Zarges. 2016)^71^	PA100	CF – oven dried	Third component – macromolecule: PPL (aqueous polyvinyl alcohol solution) 10.0 wt%	Extrusion (single-screw), extrusion (twin-screw), injection moulding [max 230 °C]	20.0–30.0
(Rahimi & Otaigbe 2017)^68^	PA6	CNC – freeze dried after modification	Surface mod. – grafting/coupling agent: aminopropyltriethoxysilane (APS) grafting on cellulose combined with *in situ* polymerized PA6 coating	Internal mixer [max 280 °C]	1.0–3.0
(Benaducci *et al.* 2016)^148^	PA66	CNF	Coated with PA6 (*via* formic acid solution)	Micro extruder, micro-injection moulding [max 280 °C]	1.0–2.0
(Semba *et al.* 2016)^77^	PA11	CNF	Surface mod. – grafting: four different reactive cationic reagents: (1) epoxy functionalized quaternary ammonium salt monomer, (2) epoxy functionalized quaternary ammonium salt polymer, (3) azetidinium ring funct. polyamide, (4) non-reactive quaternary ammonium salt [0.06–0.77 wt%]	Extrusion (twin-screw), injection moulding [max 210 °C]	10.0
(Peng *et al.* 2017)^149^	PA11	CNC – never dried	Surface mod. – esterfication: fatty side chain esterification to give dodecanoyl grafted CNC, surfactant: methyl laurate	Compression moulding [initially solvent-cast] [max 190 °C]	1–10.0

**Fig. 4:**
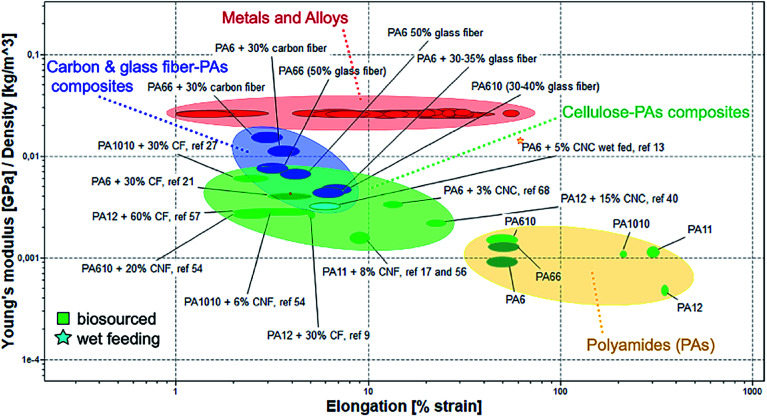
Ashby plot of specific Young's modulus as a function of the elongation for neat PAs and their different fibres composites compared with main engineering materials in use.

The Ashby plot reported in Fig. 4 compares the specific Young's modulus as a function of the elongation of the cellulose/polyamide composites with the main engineering materials in use. In the plots emerging bio-sourced polyamides are also included, for the sake of highlighting their properties in comparison with non-biosourced polyamides.

The localization in the plots of the neat biosourced polyamide suggest their possible higher deformability in comparison with the traditional PAs. It is worth notice that biosourced PAs composites reinforced with cellulosic fillers show values of Young modulus in very similar range to those reported for PAs composites reinforced with glass fibres. Interestingly, the composites based on nanocellulose achieve relatively high mechanical performance at low CNC or CNF content, indicating a good reinforcement effect. Some of these composites maintain good deformability, particularly important for allow their melt processing for some application, *e.g.* film blowing for packaging. The general overview offered by the Ashby plot underline that there is still room for improvement for interface design of nanocellulose/biosourced polyamide with improved performance, beyond their potential for secondary recycling (re-melt processability) in line with the circularity of their sustainability.

Melt processes are either batch or continuous. Lab-scale polyamide–cellulose composites *via* batch processing is often carried out in melt compounder (*e.g.* Brabender),^41,56–60^ or micro-compounder and micro-injection moulders which allow for grams scale of materials.^56,60–63^ The continuous methods, with continuous feeding, require larger amounts of materials and are less common in cellulose nanocomposites research, most likely due to limited availability of nanocelluloses and cost and time increase for the experiments. However, many studies opted for continuous processes such as twin-screw extrusion,^61,62,64–72^ which were ultimately preferred for scaling up, generally provide better component mixing and flexible modular designs (*e.g.* screw configurations, pressure control and gas/steam venting options) compared to batch-wise equipment.^31,48^

The main hurdles that prevent a successful melt-compounding of cellulosic materials with polymers in general have been summarized in a wide number of reviews to date.^2,7,21,71^ In terms of a typical melt process progression, the issues are:

(1) The irreversible hornification of cellulose materials upon drying (prior to melt processing).

(2) The non-uniform distribution/dispersion of cellulose materials in the polymer matrix.

(3) The cellulose materials thermal stability and degradation at elevated temperatures.

(4) The cellulose materials structural integrity (fibrillation) and shortening upon mechanical shearing during melt processing.

### Cellulose aggregation upon drying

Cellulose materials and in particular nanocelluloses spontaneously form tight, hydrogen-bonded networks during drying in an irreversible aggregation process often referred to as hornification,^6,42–44,47,73,74^ that occurs at temperatures as low as 40 °C.^75^ To retain the beneficial qualities of nanocellulose reinforcement, drying techniques aim to produce particulate solid materials, retaining as far as possible the nanosized structure; especially if feeding of the materials during melt processing is done by conventional means *via* a metering hopper and uniform material free-flow dosing is required. Different drying methods like oven drying, spray drying, freeze drying and supercritical drying of nanocellulose suspensions have been compared in previous studies^44^ and their advantages/disadvantages have been compiled by Ng *et al.*^49^ The influence of different drying methods on CNF was also investigated by Peng *et al.*^75^ and it was shown that the morphology as well as the surface energy can differ significantly between different drying procedures. Spray drying is generally proposed as the most technically suitable and scalable process to dry the suspensions because stable particle sizes in the nano- to micrometre scale are obtained. However, another study showed that conventional spray drying produces a compact solid structure with very low porosity compared to spray freeze drying, although the latter is more expensive.^45^ Peng *et al.*^56^ prepared composites from PA6 and different nanocelluloses (MCC, CNF and CNC) in which they characterized the size-distribution of the materials upon spray drying prior to compounding.

### Wet feeding of nanocelluloses

An approach to circumvent the self-aggregation of cellulose and nanocellulose materials upon drying is water-assisted melt processing using never-dried wet materials. Table 2 includes relevant studies on wet feeding approach during melt processing of cellulose reinforced polyamides. Typically, in such processes the aqueous filler suspension is directly fed and the water acts as a compatibilizer and plasticizer, until its evaporation during the melt process. The benefits of water-assisted production of thermoplastic nanocomposites are listed by Karger-Kocsis *et al.*^76^ This wet feeding of nanomaterials into an extrusion process leads to several advantages: (1) improved dispersion, (2) minimal degradation of cellulose, (3) surface modifications may be avoided, and (4) reduced health risks because the nanomaterials are in a slurry.^48^

Certain polyamides dissolutive behaviour in water has proven to be useful when a water-assisted melt processing route was opted for. The incorporated water in polyamides acts as a plasticizer with several advantageous exhibited material phenomena for the melt process.

**Fig. 5:**
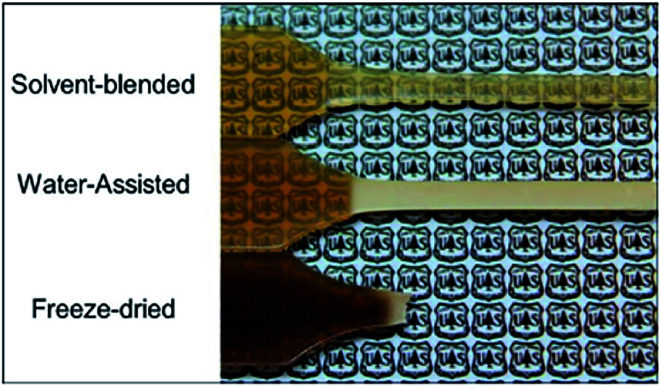
Differences in the thermal degradation of the cellulose component in PA6/CNC composites processed with three different compounding approaches, as indicated by the darkening of the specimens.^60^

**Fig. 6:**
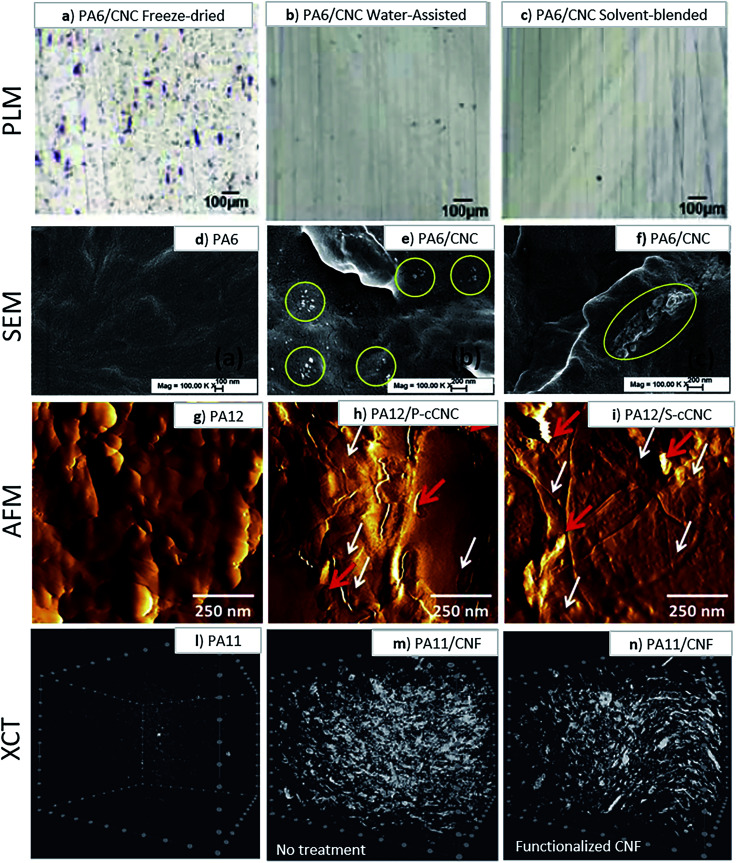
Polarized light micrographs of sections from PA6 and 5 wt% CNC composites produced by simple dry compounding of (a) freeze dried CNCs, (b) water-assisted approach and (c) solvent-blended approach, indicating an improvement in CNC dispersion with a water-assisted approach.^60^ SEM images of cryo-fractured surfaces of (d) neat PA6, (e) with 3 wt% CNC and (f) and with 3 wt% aminopropyl triethoxy silane (APS) modified CNC nanocomposites samples used to assess CNC dispersion. This figure has been adapted from ref. 69 with permission from Elsevier, copyright 2016. AFM images of thin films evaluate dispersion extent of CNCs melt compounded with PA12. (g) Neat PA12; (h) PA12 with 15 wt% phosphoric acid hydrolyzed CNCs and (i) PA12 with 15 wt% sulfuric acid hydrolyzed CNCs. This figure has been adapted from ref. 41 with permission from Wiley, copyright 2015. XCT images of (l) unfilled PA11, (m) PA11 filled with 10 wt% of unfunctionalized CNF and (n) PA11 filled with 10 wt% of functionalized CNF.^77^

These include a reduction of the polyamide glass transition temperature (*T*_g_), a lowered melt viscosity at a constant temperature, and a melting temperature and crystallization suppression due to a phenomenon known as the cryoscopic effect.^69,76^ These properties proved to be useful in water-assisted processing with a thermally sensitive and hydrophilic cellulose component, as was shown by Clemons *et al.*^60^ who successfully produced CNC reinforced PA6 composites by water-assisted melt-compounding and noted an improvement in CNC dispersion (Fig. 6a–c). They also noted a melt temperature reduction up to 45 °C (from 230 °C to 185 °C) by exceeding the 30 wt% water content which prevent the thermal degradation of the cellulosic materials (Fig. 5).

Peng *et al.*^69^ also performed water-assisted extrusion compounding of PA6 with never-dried CNCs with effective pressure and screw design (Fig. 6d–f).

### Distribution and dispersion

One of the most pervasive challenges in the preparation of composites, independent of the processing method, is the achievement of a good filler distribution and dispersion within the polymer matrix. Good distribution is defined as the fillers being distributed uniformly throughout the polymer matrix which means at any chosen volume, the amount of the fillers is the same. Good dispersion is characterized as low filler aggregation level and not significant reduction of the pristine filler aspect ratio.^56^ Composites with good distribution and dispersion of reinforcement expected to have superior final mechanical properties. When dealing with nanocellulose in particular, physical, and chemical characteristics, *e.g.* the high surface energy and hydrophilicity, promote an intrinsic tendency to aggregate. The physical challenge is the miscibility of the two polymeric materials: cellulose in the solid state into the melted polymer matrix. However, the term compatibility, which has not a defined physical meaning, is often used referring to the quality of the interactions at the two phases interface.

Several articles argue that polyamides should pair well with neat cellulose materials because of their polar nature, as well as exhibiting hydrogen bonding in their molecular structures which can lead to better compatibility between a cellulose filler and the matrix.^7,16,64,78^ It has been speculated that the cellulose with its abundant hydroxyl surface groups could form hydrogen bonds with the amines, resulting in good interfacial adhesion between cellulosic materials and polyamide matrices.^53,72^ This hydrogen bonding between the cellulose materials and the polyamides may even facilitate nucleation and promote mechanically beneficial crystallization in the polyamide matrix.^58,79^ However, despite relevant advances in the past decades, overcoming cellulose agglomeration in relatively more hydrophobic polyamides remains difficult.^50,51,80^ The quality of the distribution/dispersion is also difficult to evaluate. The inspection of cryofracture and microtome cut surfaces^81^*via* polarized light microscopy, elective dissolution of the polymer and the use of Raman imaging analysis have been proposed as tools to quantify the dispersion of nanocelluloses in thermoplastics.^82^ Instrumental methods such as AFM^41^ (Fig. 6g–i), and, in more recent studies, X-ray computed tomography^77^ (Fig. 6l–n) have also been used to evaluate the extent of nanocellulose dispersion in a polyamide. Interfacial interaction between CNF and PA11 can be also successfully assessed *via* rheological studies, in which a good interaction would be exhibited by a high melt viscosity, as was also surmised by Semba *et al.*^77^

Matching the surface chemistry of the cellulose to the polymer is a strategy commonly applied for numerous polymers *via e.g.* surface functionalization, coupling agents, non-covalent surfactant or covalently grafted hydrophobic and/or stearic moieties.^83,84^ Surface functionalization of the cellulose materials may include acetylation, esterification, silanization, silylation and glyoxalization to mention a few.^48,49^ A study of the melt processing of acetylated MFC in PA410 has been conducted by Leszczyńska *et al.*^63^ yielded better dispersion in addition to an improved thermal stability of the MFC. Another study grafted CNC *via* fatty side chain esterification to improve the CNC interfacial interaction and dispersibility in PA11.^85^ Silyl coupling agents, employed in both gas and liquid phase, were used to surface functionalize CF and CNC with subsequent melt processing in polyamides with promising results.^62,68^ Surfactants have been used in extrusion melt processes to improve CNC dispersion in many other polymeric matrices before,^85^ and a study by Peng *et al.* used a methyl laurate surfactant as a plasticizer for the processing of CNC in PA11. A treatment with cation reagents can change the cellulose surface charge from negative to positive, and is a strategy which was used in the melt processing of CNF in PA11 and PA12.^66,77,86^ An example of coupling agents are silanes, which are commonly employed in the composite industry,^87,88^ and an aminosilane has been used on cellulose fibres for melt processing with PA12,^62^ and on CNC in PA6.^68,89^ Cellulose materials can also be coated with a hydrophobic polymer to avoid their aggregation during drying and to improve their distribution/dispersion in the polymer matrix, as was done by Corrêa *et al.* who coated CNC in PA6 and Zarges *et al.* who coated CF in PVA prior to melt compounding in polyamide matrices.^71,72,90,91^

### Thermal degradation of cellulose

Depending on their source and processing, the decomposition of cellulosic fibres is typically assessed by thermogravimetrical analysis (TGA, under nitrogen atmosphere at heating rate of 10 °C min^−1^) and occurs in the range of 150–450 °C. This corresponds to the decomposition of glycosylic units leading to a breakdown of the structure and formation of low molecular weight gaseous products like H_2_O, CO_2_, alkanes, and other hydrocarbon derivatives.^92^ The thermal degradation of celluloses is a three step process: the first step being the elimination of water; the second – and central step – begins from approximately 250 °C and is the advanced depolymerization of the cellulose resultant from the dehydration and decomposition of glycosyl to form char; and a third step, above 425 °C, assigned to further degradation of charred residue into gaseous products.^93^ The choice of cellulose material and acknowledging its thermal stability is of importance, particularly for melt-compounding processes exceeding 200 °C, as in the case of polyamides. Sulfuric acid hydrolyzed CNCs are particularly sensitive because the production process incorporates sulphate groups on the cellulose crystal surface which are thermally unstable.^41,93^ Hydrochloric acid hydrolysis may be used instead but resultant CNCs tend to aggregate more easily in the aqueous state, due to different surface charge characteristics, and are often difficult to re-disperse. On the other hand, CNCs prepared by phosphoric acid, which are less common, were found to exhibit acceptable dispersion in polymers and a much higher thermal stability than sulfuric acid prepared CNCs.^94^ This was the case for a study focused on PA6/CNC melt processing in which the authors advocated the use of phosphoric acid prepared CNCs as pertinent to their good results.^41^

Another approach are the various cellulose surface modifications (mentioned in the previous section) which can serve the added purpose of improving cellulose thermal stability. It is possible to coat the cellulose material *via* either chemical or physical wrapping with a macromolecule or surfactant, which may impart improved thermal stability to the cellulose. This is exemplified in studies in which modified CNFs with ionically adsorbed quaternary ammonium salts bearing long alkyl chains, through simple aqueous adsorption, resulted in improved thermal stability of nanocelluloses processed with PA12.^77,95^ Studies involving the coating of CNC with dissolved PA6 (followed by drying the materials and then melt processing),^72^ and the coating of CF with PVA provide cellulose with increased thermal stability.^71^ A more intricate attempt used a multi-step process to incorporate CNCs in PA6 consisted of an *in situ* polymerization of caprolactam monomer (to yield PA6) in the presence of silane surface modified CNCs followed by melt extrusion.^68^ The use of various melt-temperature profile control and processing techniques to lower the overall processing temperature is a route that has been explored for several polyamides.^70,71^ Additives such as plasticizers, ceramic powders and inorganic halide salts have also been used separately and in combination as attempts to decrease the processing temperature and control melt viscosity for polyamides.^65^ Recently, the addition of LiCl has been confirmed to be a way to suppress the melting point of polyamides.^96,97^ A number of studies have also motivated water-assisted or liquid-mediated techniques as a means to circumvent cellulose thermal degradation issues in melt processes.^60,69,76^

### Cellulose structural integrity upon mechanical shearing

**Fig. 7:**
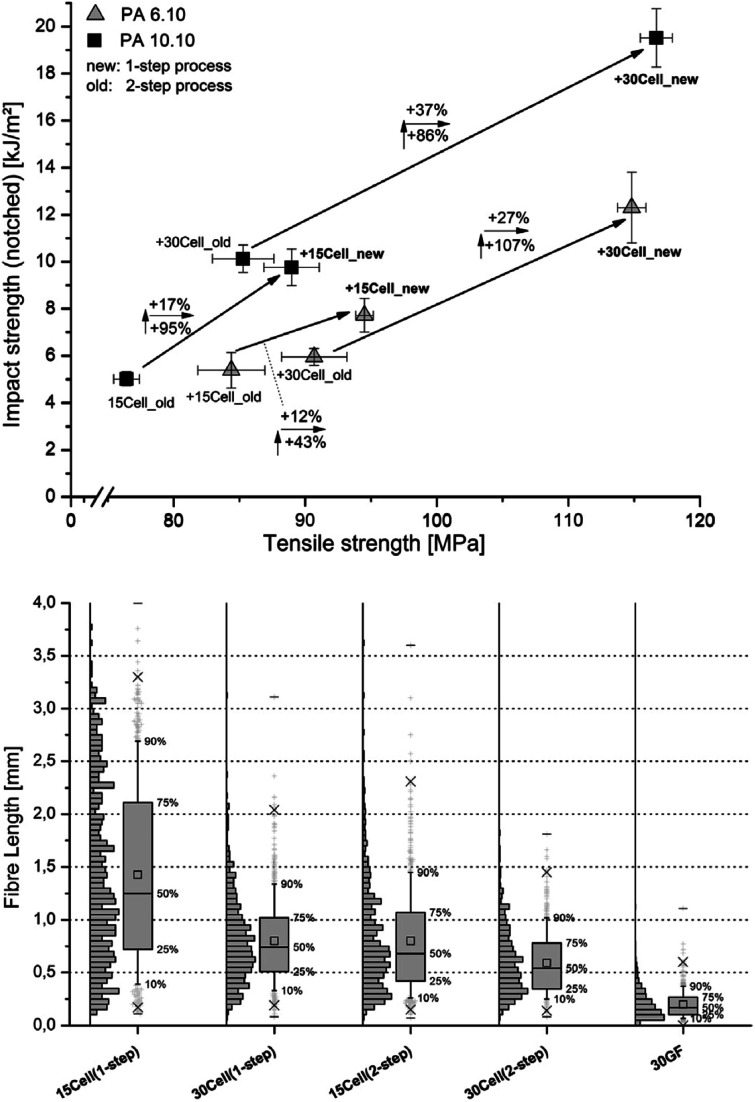
Influence of the compounding process on the impact strength and tensile strength of PA 6.10 and PA 10.10 composites with 30% cellulose fibres content processed using a twin-screw extruder (top image). Fibre length of PA 6.10 composites with various fibres (cellulose at 15 and 30 wt% *vs.* 30 wt%) glass fibres (GF) processed by different compounding methods – box plot illustration (bottom image). This figure has been adapted from ref. 99 with permission from Elsevier, copyright 2014.

Melt processes that involve particularly high shear melt mixing rates can have an adverse effect on the structural integrity of cellulosic materials, however this aspect is often overlooked in the research. Evaluation of the shear melt mixing influence can be determined through various imaging observations, *e.g.* the length and aspect ratios of nanocelluloses before and after processing. The mechanical degradation is exhibited by the reduction in the nanocellulose length, affecting the overall mechanical and stress-transfer properties of the reinforcement.^21,51,71^ A selective dissolution of the polymer matrix (by using an Soxhlet apparatus) allows to recover the cellulose materials from the composites (after melt processing) for further morphological analysis. Blends of formic acid (FA) and methylene chloride (DCM) have been used successfully to dissolve polyamide composites to obtain reinforcing cellulose fibers for morphological characterization.^98^ Feldmann *et al.*^99^ reported that glass fibers break more often when preparing the specimen using the injection molding process than the cellulose fibres because of their higher stiffness and lower elongation at break (Fig. 7, bottom image). Moreover, they showed that the processing steps and the amount of cellulose incorporated into the polymeric matrix affect the structural integrity of the cellulose fibres. In fact, composites processed by the single step injection moulding and thus, avoiding the previous extrusion process, showed less damaged fibres, resulting longer than those submitted to the two-step process. Furthermore, increasing the amount of fibres in the polymeric matrix the damage of the fibres is higher, resulting in shorter fibres (Fig. 7, bottom image). This behaviour is reflected in the mechanical properties of the composites as it is possible to observe in Fig. 7 (top image). Composites obtained with lower thermal and mechanical stressed fibres both due to the processing method or thanks to the amount of filler used as well, show high mechanical performance.^99^

### Reactive melt processing

An approach relatively unexplored is reactive melt processing. Reactive melt-processes introduce a reactive agent(s) at chosen points during a conventional melt-process. The reaction initiation can occur while homogenizing the materials in the melt and is usually allowed sufficient time for completion.^100^ Such chemical reactions may include *e.g.* polymerizations, grafting, branching, controlled cross-linking, coupling and functionalization of the processed materials.^101^ In a typical reactive extrusion process, the reactants can be fed to the extruder through normal means *via* the feed hopper or injected into specific points of the barrel. This allows the use of various liquid or gaseous reactants and the tailoring of intricate reaction sequences. An early attempt in 1985 utilized a two liquid component reaction injection moulding process to produce cellulose fibre reinforced *in situ* polymerized PA6.^102^ An interesting review focused on the reactive extrusion of biodegradable polymers has been conducted by Raquez *et al.*^101^

### Piezoelectricity as potential emerging application of PAs

Thanks to its interesting properties, bio-PA are used in many field of application such as automotive, construction, electronics, food and medical industries ranging from cable ties, fuel line applications for the automotive, metal coatings, advanced medical materials for prosthetic devices, 3D printing, additive manufacturing, filaments such as bristles for toothbrushes, shoe soles for high-tech sport, cable housings, *etc.*^11^ In this section a potential emerging application will be discussed.

The continuous growing demand of renewable energy solutions for portable smart electronic devices, experimented in the last two decades, has stimulated the development of advanced energy harvesting technologies from wasted energy sources. It could represent a promising alternative to fossil fuel.^103^ In this context, devices based on piezoelectric materials can be a challenging alternative to convert mechanical energies into electricity for energetically autonomous wireless and electronic devices.^104^ Indeed, piezoelectric materials are able to respond to both mechanical and electrical stimuli by producing energy or deforming mechanically depending on the nature of the stimulus. It is for this reason that these materials are of great interest and already have many applications in several areas such as piezoelectric transducers, sensors, actuators and energy harvesters.^105,106^ Piezoelectric energy harvesters (PEHs) are regarded as promising independent renewable power sources for low-power electronic devices such as wireless sensors, portable devices, and medical implants.^107^

Currently, commercial PEHs use piezoelectric ceramics with high piezoelectric coefficients providing excellent energy performance. However, these ceramics are still expensive and not easy scalable for industrial production of PEHs devices.^108^ Moreover, the increasing demand for flexible, translucent or transparent smart devices opens up for piezoelectric polymers which have lower piezoelectric coefficients, but they show advantages in terms of cost and large-scale processability.^109^

The tensile piezoelectricity in stretched and poled films of polyvinylidene fluoride (PVDF) was first demonstrated by Kawai in 1969.^110^ This discovery triggered widely spread investigations on the pyro-, piezo-, and ferroelectricity of PVDF, its copolymers, nylons, and other polymers for subsequent years.^111,112^

PAs are characterized by a repeating unit of [HN–(CH_2_)_*x*_–CO]_*n*_, with odd or even number of carbons atoms between the amide groups. Odd numbered PAs contain pairs of –NH and –C=O groups aligned in the same direction that are able to form stable dipole moments, which leads to a polar structure that exhibit their observed ferroelectric, piezoelectric and pyroelectric behaviour.^113,114^ In comparison, these groups in even numbered PAs are aligned in an alternating way, leading to the net cancelation of the dipoles along the polymeric chains.^115^

In 1991, Takase *et al.*^116^ studied the variation of the piezoelectric response of PA11 and PA7 with temperature, compared to that of PVDF. They observed the highest values of piezoelectric strain constant (*d*_31_ = 14 pC N^−1^ and 17 pC N^−1^ for PA11 and for PA7, respectively), stress constant (*e*_31_ = 21 mC m^−2^ and 27 mC m^−2^ for PA11 and for PA7, respectively) and electromechanical coupling coefficient (*k*_31_ = 0.054 for PA11 and 0.049 for PA7) of PA11 and PA7 films at temperature ranges from 100 to 200 °C.^103^ This behaviour suggested that the orientation of dipoles depends on the temperature, so that above glass transition temperature (*T*_g_), the mobility of the polymeric chains is high, and they are more sensitive to orientation induced by the electric field applied. In the same work, Takase *et al.*^116^ observed that the cooling rate have also a notable influence on the piezoelectric constant, since the crystal structures depends on the processing condition. Using the same polarization method, if the material is quenched, the formation of the γ phase is favoured while a slow cooling rate leads to the formation of the α phase.

Although all PA11 have a polar crystal structure due to its dipole's orientations, specific crystalline phase types can maximize the electric polarization.^114^ It is known that γ crystalline phase has the best piezoelectric response in PA11 even if in literature is reported that δ′ phase may also contribute.^117,118^

In 1993, B. Z. Mei *et al.* found that the remanent polarization and the coercive field of odd PAs series (PA5, PA7, PA9 and PA11) increase linearly with dipole density as does the melting point. It is important to specify that, decreasing the number of CH_2_ groups in PAs chains, the concentration of amide groups increases as well as the dipole density. Their work showed also that both uniaxially oriented and unoriented odd-numbered PAs exhibit ferroelectric hysteresis behaviour but those uniaxial oriented show higher ferroelectric response.^119–121^

Between odd PAs, PA11 can be synthesized by renewable resources (castor oil), thus contributing to have a much smaller carbon footprint and consequently decreasing the environmental impact of PEHs devices.^122^ Apart from the excellent properties such as high flexibility, optical transparency, relative cheapness, similarly to other biosourced polymers, PA11 has also other advantages, non-toxicity and biocompatibility. In this context, PA11 could be a good alternative for implants and biomedical health monitoring systems, which require devices to be biocompatible.^123^

**Fig. 8:**
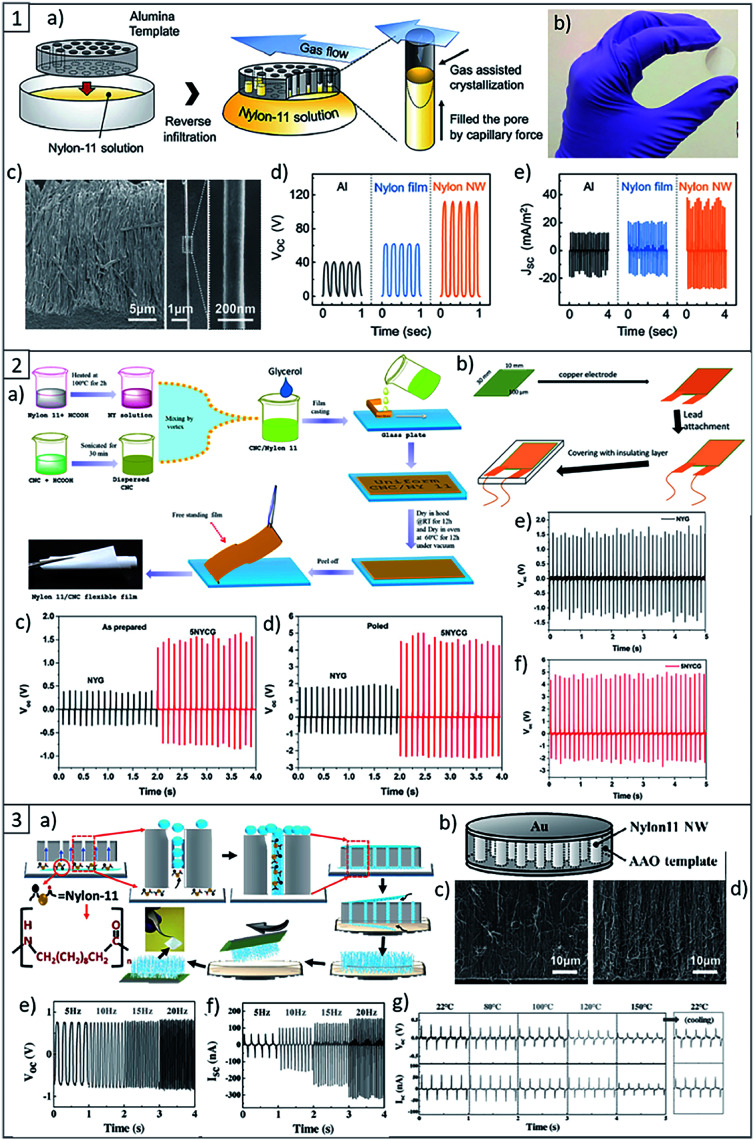
(1) (a) Scheme of the nanowire fabrication procedure. (b) Photographs of a fabricated nanowire filled AAO template. (c) SEM images of template-freed nanowires and a single strand of PA nanowire, respectively. (d) Open-circuit output voltages and (e) short-circuit output current densities of the triboelectric generators with different combinations of materials. This figure has been adapted from ref. 125 with permission from Royal Society of Chemistry, copyright 2017. (2) (a) Scheme of PA11 and Its Composite Films preparation. (b) Scheme of piezoelectric nanogenerators fabrication. (c) Open circuit voltage of PA11 nanogenerator. (d) Open circuit voltage of PA11 + 5 wt% of CNC nanogenerator. (e) Output voltage of poled PA11 and (f) PA11 + 5 wt% CNC nanogenerator after 3 months of storage. This figure has been adapted from ref. 126 with permission from American Chemical Society, copyright 2019. (3) (a) Scheme of the growth mechanism of PA11 nanowires inside AAO template *via* capillary wetting. (b) Scheme of the PA11 nanowire-based piezoelectric nanogenerator. (c) Cross-sectional SEM images of the PA11 nanowire generator before testing and (d) after testing. (e) Open circuit voltage of the PA11 nanowire-based nanogenerator. (f) Short circuit current output of the PA11 nanowire-based nanogenerator. (g) High-temperature electrical output data of the PA11 nanowire-based nanogenerator recorded at room temperature (22 °C) and at temperatures up to 150 °C. This figure has been adapted from ref. 124 with permission from Wiley, copyright 2017.

With the aim to increase the piezoelectric response of PA11, in literature are reported several strategies, such as different methods to process the material, *i.e.* the fabrication of self-poled nanowires (Fig. 8(1) and (3)), which are highly flexible, lightweight and sensitive to small vibrations, as well as mechanical stretching and annealing to increase the dipoles orientation and the degree of crystallinity of the piezoelectric crystalline phase of PA.^104,124^

Another method reported in literature to improve the piezoelectric properties of PA11, is the incorporation of nanofillers such as piezoelectric lead zirconate titanate,^127^ barium titanate,^128^ layered silicates^129,130^ and CNCs.^126^

Cellulose is classified as natural piezoelectric material and its piezoelectric response have been studied in the last years, becoming a suitable material for energy harvesting device as well as storage application. The shear piezoelectricity of cellulose-based biopolymers such as wood, amylase, chitin, and starch can be comparable to that of quartz crystal.^131^

In 2012, Csoka *et al.*^132^ reported the piezoelectric response of ultrathin films of aligned CNCs. Their piezoelectric properties were ascribed to the collective contribution of the asymmetric crystalline structure of the cellulose crystals, showing a shear piezoelectric constant value comparable to that of a reference piezoelectric metal oxide. Some year after, Rajala *et al.*^133,134^ prepared piezoelectric sensors from CNF film and investigated their piezoelectric response. They found sensitivity values between 4.7 and 6.4 pC N^−1^ in ambient conditions. After comparing these results with PVDF-based sensor devices, the authors suggested CNF-based materials as a suitable precursor material for disposable piezoelectric sensors, actuators and energy generators with potential applications in the fields of electronics, sensors, and biomedical diagnostics.^103^

More recently, F. Ram *et al.*^126^ reported the fabrication of a flexible piezoelectric energy harvester based on PA11 and CNCs by a solution casting process. They showed for the first time in literature the possibility to enhance the electroactive γ phase in PA11 using CNCs, resulting in the increase of the piezoelectric performance of the device (Fig. 8(2)). In fact, the incorporation of low concentration of CNCs, *i.e.* 2–5 wt% in the PA11 matrix resulted in the almost complete transition of α-phase to the polar γ-phase. As a result of the best formulation, energy harvesting devices made from PA11 reinforced with 5 wt% of CNCs showed about 2.6 times higher output voltage as compared to the devices composed by the neat matrix under similar impact conditions, and the effect was durable over 800 cycles.^126^ Considering the high piezoelectric performance of PA11 and CNCs, together with their low cost, flexibility, durability, and the possibility to process this material by an environmentally-friendly, sustainable and scalable method such as the reactive extrusion lead to consider them as promising materials for potential advanced applications in self powering sensors and powering of other small electronics.

## Conclusions and perspectives

Biosourced polyamides are promising engineering thermoplastics fully or partially derived from renewable feedstock. As traditional PAs, they exhibit high mechanical strength and thermal performance showing added processing advantages and better properties. The general main challenges of incorporating cellulosic materials in polymeric matrices have largely impeded the successful preparation of nanocellulose–polyamide composites *via* large-scale melt processing methods. To our knowledge, the number of reported studies involving the manufacture of cellulose–polyamide composites at a large-scale is still limited but has gained interest in the past five years being a cost efficient, sustainable, and organic solvent-free method. Wet feeding has been introduced as a means to overcome dispersion and nanocellulose agglomeration issues as well as its thermal degradation thanks to the plasticizer effect of water which is able to reduce PAs melting temperature. Moreover, water assisted compounding might enhance the degree of crystallinity which yielded further mechanical property benefits. Furthermore, as discussed previously, biosourced PAs are good candidates for the development of advanced energy harvesting devices based on piezoelectric materials, being a challenging alternative to convert mechanical wasted energies into electricity as a promising alternative to fossil fuel. Considering the high piezoelectric performance of PAs based composites reinforced with cellulose, together with their low cost, excellent mechanical properties, durability, and the possibility to process this material by a solvent-free, sustainable and scalable method such as reactive extrusion and wet feeding, this materials are considered as promising materials for potential advanced applications in self powering sensors and powering of other small electronics.

## Conflicts of interest

There are no conflicts to declare.

## Supplementary Material

## References

[cit1] Geyer R., R Jambeck J., Law K. L. (2017). Sci. Adv..

[cit2] Moon R. J., Martini A., Nairn J., Simonsen J., Youngblood J. (2011). Chem. Soc. Rev..

[cit3] Klemm D., Heublein B., Fink H. P., Bohn A. (2005). Angew. Chem., Int. Ed..

[cit4] Miao C., Hamad W. Y. (2013). Cellulose.

[cit5] Sehaqui H., Allais M., Zhou Q., Berglund L. A. (2011). Compos. Sci. Technol..

[cit6] Eichhorn S. J. (2011). Soft Matter.

[cit7] Mariano M., El Kissi N., Dufresne A. (2014). J. Polym. Sci., Part B: Polym. Phys..

[cit8] Gilbert M. , Aliphatic Polyamides, in Brydson's Plastics Materials, Elsevier, 2017, vol. 5, pp. 487–511

[cit9] Kutz M. , 9.5.2.1 Polyamide 6/6 and 6 (PA6, PA6/6), in Mechanical Engineers' Handbook, Volume 1 - Materials and Engineering Mechanics, John Wiley & Sons, 4th edn, 2015

[cit10] Melton G. H. , Peters E. N. and Arisman R. K., 2 - Engineering Thermoplastics A2 - Kutz, Myer. Applied Plastics Engineering Handbook 2011, pp. 7–21

[cit11] (b) Ecologic Institute , Top Emerging Bio-based Products, their Properties and Industrial Applications, Berlin, 2018

[cit12] Klason C., Kubát J., Strömvall H.-E. (1984). Int. J. Polym. Mater. Polym. Biomater..

[cit13] (a) Research G. V. , Bio-Polyamide Market Size, Share & Trends Analysis Report By Product (PA 6, PA 66, Specialty Polyamide) By Application, By End use (Textile, Automotive, Coating, Sports, Industrial, Electronics), And Segment Forecasts, 2018 - 2025, 2017, p. 127

[cit14] Francisco D. L., Paiva L. B., Aldeia W. (2018). Polym. Compos..

[cit15] Intelligence M. , Polyamides Market - Growth, Trends, and Forecast (2020–2025), 2019, p. 106

[cit16] Zhu J. H. , Kiziltas A., Lee E. C. and Mielewski D., Spe Acce, 2015

[cit17] Nair S. S., Ramesh C., Tashiro K. (2006). Macromolecules.

[cit18] Panaitescu D. M., Gabor R. A., Frone A. N., Vasile E. (2015). J. Nanomater..

[cit19] Zhang Q., Mo Z., Liu S., Zhang H. (2000). Macromolecules.

[cit20] De Assis C. A., Houtman C., Phillips R., Bilek E. M. T. M. T., Rojas O. J., Pal L., Peresin M. S., Jameel H., Gonzalez R. (2017). Biofuels, Bioprod. Biorefin..

[cit21] Dufresne A. (2018). Philos. Trans. R. Soc., A.

[cit22] Abitbol T., Rivkin A., Cao Y., Nevo Y., Abraham E., Ben-Shalom T., Lapidot S., Shoseyov O. (2016). Curr. Opin. Biotechnol..

[cit23] Habibi Y., Lucia L. A., Rojas O. J. (2010). Chem. Rev..

[cit24] Peng B. L., Dhar N., Liu H. L., Tam K. C. (2011). Can. J. Chem. Eng..

[cit25] Börjesson M. and Westman G., Fundamental Aspects and Current Trends, InTech, 2015, vol. 2, p. 64

[cit26] Cellulose Nanocrystals technology|CelluForce, https://www.celluforce.com/en/products/cellulose-nanocrystals/

[cit27] Nanotech Magazine , Issue 51: Cellulose nanocrystals market, automotive nanocomposites, all the latest nanotech business news, in Issue 51: The Market for Nanocrystalline Cellulose (NCC)/Cellulose Nanocrystal (CNC), 2018, pp. 4–8

[cit28] Chinga-Carrasco G. (2011). Nanoscale Res. Lett..

[cit29] Neagu R. C., Gamstedt E. K., Berthold F. (2005). J. Compos. Mater..

[cit30] Brinchi L., Cotana F., Fortunati E., Kenny J. M. (2013). Carbohydr. Polym..

[cit31] Lee K. Y., Aitomäki Y., Berglund L. A., Oksman K., Bismarck A. (2014). Compos. Sci. Technol..

[cit32] Lo Re G. and Sessini V., Wet feeding approach for cellulosic materials/PCL Biocomposites, in ACS Symposium Series - Biomass extrusion and reaction technologies: Principles to practices and future potential, American Chemical Society, 2018, vol. 1304, pp. 209–226

[cit33] Poletto M., Pistor V., Zeni M., Zattera A. J. (2011). Polym. Degrad. Stab..

[cit34] Oliaei E., Lindén P. A., Wu Q., Berthold F., Berglund L., Lindström T. (2020). Cellulose.

[cit35] Jörg P., Thoralf G., Wolfgang B. (2016). Nord. Pulp Pap. Res. J..

[cit36] Quiévy N., Jacquet N., Sclavons M., Deroanne C., Paquot M., Devaux J. (2010). Polym. Degrad. Stab..

[cit37] Kargarzadeh H., Mariano M., Huang J., Lin N., Ahmad I., Dufresne A., Thomas S. (2017). Polymer.

[cit38] Klemm D., Cranston E. D., Fischer D., Gama M., Kedzior S. A., Kralisch D., Kramer F., Kondo T., Lindström T., Nietzsche S., Petzold-Welcke K., Rauchfuß F. (2018). Mater. Today.

[cit39] Spinella S., Lo Re G., Liu B., Dorgan J., Habibi Y., Leclère P., Raquez J.-M., Dubois P., Gross R. A. (2015). Polymer.

[cit40] Roman M., Winter W. T. (2004). Biomacromolecules.

[cit41] Nicharat A., Sapkota J., Weder C., Johan Foster E. (2015). J. Appl. Polym. Sci..

[cit42] Newman R. H. (2004). Cellulose.

[cit43] Idström A., Brelid H., Nydén M., Nordstierna L. (2013). Carbohydr. Polym..

[cit44] Peng Y., Gardner D. J., Han Y. (2012). Cellulose.

[cit45] Kamal M. R., Khoshkava V. (2015). Carbohydr. Polym..

[cit46] Vilela C. , Pinto R. J. B., Figueiredo A. R. P., Neto C. P., Silvestre A. J. D. and Freire C. S. R., 1 Development and applications of cellulose nanofibres based polymer nanocomposites, 2017, pp. 1–65

[cit47] Santos F. A. d., Iulianelli G. C. V., Tavares M. I. B. (2016). Mater. Sci. Appl..

[cit48] Oksman K., Aitomäki Y., Mathew A. P., Siqueira G., Zhou Q., Butylina S., Tanpichai S., Zhou X., Hooshmand S. (2016). Composites, Part A.

[cit49] Ng H. M., Sin L. T., Bee S. T., Tee T. T., Rahmat A. R. (2017). Polym.-Plast. Technol. Eng..

[cit50] Kargarzadeh H., Huang J., Lin N., Ahmad I., Mariano M., Dufresne A., Thomas S., Gałęski A. (2018). Prog. Polym. Sci..

[cit51] Ferreira F. V., Dufresne A., Pinheiro I. F., Souza D. H. S., Gouveia R. F., Mei L. H. I., Lona L. M. F. (2018). Eur. Polym. J..

[cit52] Dufresne A. (2013). Mater. Today.

[cit53] Qua E. H., Hornsby P. R. (2011). Plast., Rubber Compos..

[cit54] Heshmati V., Kamal M. R., Favis B. D. (2018). Eur. Polym. J..

[cit55] Garcia-Ramirez M., Cavaillé J. Y., Dupeyre D., Péguy A. (1994). J. Polym. Sci., Part B: Polym. Phys..

[cit56] Peng Y., Gardner D. J., Han Y. (2015). Cellulose.

[cit57] Panaitescu D. M., Frone A. N., Nicolae C. (2013). Eur. Polym. J..

[cit58] Lu J. Z., Doyle T. W., Li K. (2007). J. Appl. Polym. Sci..

[cit59] Kiziltas A., Gardner D. J., Han Y., Yang H.-S. (2014). J. Polym. Environ..

[cit60] Clemons C. , in Annual Technical Conference - ANTEC, Conference Proceedings, 2015, pp. 430–434

[cit61] Winata H. , Turng L.-S., Caulfield D. F., Kuster T., Spindler R. and Jacobson R., in Annual Technical Conference - ANTEC, Conference Proceedings, Nashville, 2003, vol. 1, pp. 701–705

[cit62] Paunikallio T., Suvanto M., Pakkanen T. T. (2006). J. Appl. Polym. Sci..

[cit63] Leszczyńska A., Kiciliński P., Pielichowski K. (2015). Cellulose.

[cit64] Yousefian H., Rodrigue D. (2016). Polym. Compos..

[cit65] Xiaolin X. , Cellulose Fiber Reinforced Nylon 6 or Nylon 66, 2008, pp. 1–228

[cit66] Semba T., Ito A., Kitagawa K., Nakatani T., Yano H., Sato A. (2014). J. Appl. Polym. Sci..

[cit67] Rohner S., Humphry J., Chaléat C. M., Vandi L. J., Martin D. J., Amiralian N., Heitzmann M. T. (2018). Cellulose.

[cit68] Rahimi S. K., Otaigbe J. U. (2017). Polymer.

[cit69] Peng J., Walsh P. J., Sabo R. C., Turng L.-S., Clemons C. M. (2016). Polymer.

[cit70] Jacobson R. , Caulfield D., Sears K., Underwood J. and Caufield J., The Sixth International Conference on Woodfiber-Plastic Composites, 2001, pp. 127–133

[cit71] Feldmann M., Heim H. P., Zarges J. C. (2016). Composites, Part A.

[cit72] Corrêa A. C., de Morais Teixeira E., Carmona V. B., Teodoro K. B. R., Ribeiro C., Mattoso L. H. C., Marconcini J. M. (2014). Cellulose.

[cit73] Salmén L. (2018). Cellulose.

[cit74] Diniz J. M. B. F. , Gil M. H. and Castro J. A. A. M., Hornification—its origin and interpretation in wood pulps, 2004, vol. 37, pp. 489–494

[cit75] Peng Y., Gardner D. J., Han Y., Cai Z., Tshabalala M. A. (2013). J. Colloid Interface Sci..

[cit76] Karger-Kocsis J., Kmetty Á., Lendvai L., Drakopoulos S. X., Bárány T. (2015). Materials.

[cit77] Semba T., Taguma K., Tawara M., Ito A., Kitagawa K., Sato A., Yano H. (2016). Nihon Reoroji Gakkaishi.

[cit78] Armioun S., Panthapulakkal S., Scheel J., Tjong J., Sain M. (2016). J. Appl. Polym. Sci..

[cit79] Kiziltas A., Nazari B., Gardner D. J., Bousfield D. W. (2014). Polym. Eng. Sci..

[cit80] Gardner D. J., Oporto G. S., Mills R., Samir M. A. S. A. (2008). J. Adhes. Sci. Technol..

[cit81] Gohn A. M., Seo J., Ferris T., Venkatraman P., Foster E. J. (2019). Thermochim. Acta.

[cit82] Lewandowska A. E., Eichhorn S. J. (2016). J. Raman Spectrosc..

[cit83] Natterodt J. C., Sapkota J., Foster E. J., Weder C. (2017). Polymer. Biomacromolecules.

[cit84] Lin N., Huang J., Dufresne A. (2012). Nanoscale.

[cit85] Bondeson D., Oksman K. (2007). Compos. Interfaces.

[cit86] Alila S., Boufi S., Belgacem M. N., Beneventi D. (2005). Adsorption of a cationic surfactant onto cellulosic fibers I. Langmuir.

[cit87] Wu H. F., Dwight D. W., Huff N. T. (1997). Compos. Sci. Technol..

[cit88] Lee J. A., Yoon M. J., Lee E. S., Lim D. Y., Kim K. Y. (2014). Macromol. Res..

[cit89] Kashani Rahimi S., Otaigbe J. U. (2016). Polymer.

[cit90] Lin N., Dufresne A. (2013). Macromolecules.

[cit91] Azouz K. B., Ramires E. C., Van Den Fonteyne W., El Kissi N., Dufresne A. (2012). ACS Macro Lett..

[cit92] Elanthikkal S., Gopalakrishnapanicker U., Varghese S., Guthrie J. T. (2010). Carbohydr. Polym..

[cit93] Roman M., Winter W. T. (2004). Biomacromolecules.

[cit94] Camarero Espinosa S., Kuhnt T., Foster E. J., Weder C. (2013). Biomacromolecules.

[cit95] Nagalakshmaiah M., El Kissi N., Dufresne A. (2016). ACS Appl. Mater. Interfaces.

[cit96] Xu S., Sun L., He J., Han H., Wang H., Fang Y., Wang Q. (2018). Polym. Compos..

[cit97] Amintowlieh Y., Sardashti A., Simon L. C. (2012). Polym. Compos..

[cit98] Oliver-Ortega H., Granda L. A., Espinach F. X., Mendez J. A., Julian F., Mutjé P. (2016). Compos. Sci. Technol..

[cit99] Feldmann M., Bledzki A. K. (2014). Compos. Sci. Technol..

[cit100] Xanthos M., Dagli S. S. (1991). Polym. Eng. Sci..

[cit101] Raquez J. M., Narayan R., Dubois P. (2008). Macromol. Mater. Eng..

[cit102] Zadorecki P., Abbås K. B. (1985). Polym. Compos..

[cit103] Mishra S., Unnikrishnan L., Nayak S. K., Mohanty S. (2019). Macromol. Mater. Eng..

[cit104] Whiter R. A., Narayan V., Kar-Narayan S. (2014). Adv. Energy Mater..

[cit105] Zhou M., Al-Furjan M. S. H., Zou J., Liu W. (2018). Renewable Sustainable Energy Rev..

[cit106] Yang Z., Zhou S., Zu J., Inman D. (2018). Joule.

[cit107] Chorsi M. T., Curry E. J., Chorsi H. T., Das R., Baroody J., Purohit P. K., Ilies H., Nguyen T. D. (2019). Adv. Mater..

[cit108] Narita F., Fox M. (2018). Adv. Eng. Mater..

[cit109] Usher T. D., Cousins K. R., Zhang R., Ducharme S. (2018). Polym. Int..

[cit110] Kawai H. (1969). Jpn. J. Appl. Phys..

[cit111] Mohebbi A., Mighri F., Ajji A., Rodrigue D. (2018). Adv. Polym. Technol..

[cit112] Ramadan K. S., Sameoto D., Evoy S. (2014). Smart Mater. Struct..

[cit113] Mathur S. C., Scheinbeim J. I., Newman B. A. (1984). J. Appl. Phys..

[cit114] Jing Q., Kar-Narayan S. (2018). J. Phys. D: Appl. Phys..

[cit115] Harrison J., Ounaies Z. (2002). Encycl. Polym. Sci. Technol..

[cit116] Takase Y., Lee J. W., Scheinbeim J. I., Newman B. A. (1991). Macromolecules.

[cit117] Scheinbeim J. I. (1981). J. Appl. Phys..

[cit118] Zhang Z., Litt M. H., Zhu L. (2016). Macromolecules.

[cit119] Mei B. Z., Scheinbeim J. I., Newman B. A. (1993). Ferroelectrics.

[cit120] Wu S. L., Scheinbeim J. I., Newman B. A. (1999). J. Polym. Sci., Part B: Polym. Phys..

[cit121] Mathur S. C., Newman B. A., Scheinbeim J. I. (1988). J. Polym. Sci., Part B: Polym. Phys..

[cit122] Kim H. C., Mun S., Ko H.-U., Zhai L., Kafy A., Kim J. (2016). Smart Mater. Struct..

[cit123] Gaur A., Tiwari S., Kumar C., Maiti P. (2019). Nanoscale Adv..

[cit124] Datta A., Choi Y. S., Chalmers E., Ou C., Kar-Narayan S. (2017). Adv. Funct. Mater..

[cit125] Choi Y. S., Jing Q., Datta A., Boughey C., Kar-Narayan S. (2017). Energy Environ. Sci..

[cit126] Ram F., Radhakrishnan S., Ambone T., Shanmuganathan K. (2019). ACS Appl. Polym. Mater..

[cit127] Li K., Wang H., Ding A. (2010). Polym. Sci., Ser. B.

[cit128] Capsal J.-F., Dantras E., Laffont L., Dandurand J., Lacabanne C. (2010). J. Non-Cryst. Solids.

[cit129] Li Y., Iwakura Y., Shimizu H. (2008). J. Nanosci. Nanotechnol..

[cit130] Leveque M., Douchain C., Rguiti M., Prashantha K., Courtois C., Lacrampe M. F., Krawczak P. (2017). Int. J. Polym. Anal. Charact..

[cit131] Khan A., Abas Z., Kim H. S., Kim J. (2016). Sensors.

[cit132] Csoka L., Hoeger I. C., Rojas O. J., Peszlen I., Pawlak J. J., Peralta P. N. (2012). ACS Macro Lett..

[cit133] Rajala S. , Vuoriluoto M., Rojas O. J., Franssila S. and Tuukkanen S., in Piezoelectric sensitivity measurements of cellulose nanofibril sensors, IMEKO XXI World Congress, Proceedings, August 30-September 4, 2015, Prague, Czech Republic, 2015

[cit134] Rajala S., Siponkoski T., Sarlin E., Mettänen M., Vuoriluoto M., Pammo A., Juuti J., Rojas O. J., Franssila S., Tuukkanen S. (2016). ACS Appl. Mater. Interfaces.

[cit135] Chen J., Gardner D. J. (2008). Polym. Compos..

[cit136] De Arcaya P. A., Retegi A. A., Arbelaiz A., Kenny J. M. J. M., Mondragon I. I. (2009). Polym. Compos..

[cit137] Feldmann M., Bledzki A. K. (2014). Compos. Sci. Technol..

[cit138] Zierdt P., Kulkarni G., Theumer T. (2017). Macromol. Symp..

[cit139] Fernandes F. C., Gadioli R., Yassitepe E., De Paoli M. A. (2017). Polym. Compos..

[cit140] Yousefian H., Rodrigue D. (2017). J. Cell. Plast..

[cit141] Xu S., Fang Y., Yi S., He J., Zhai X., Song Y., Wang H., Wang Q. (2018). Polym. Test..

[cit142] Beg M. D. H. , Islam M. R., Mamun A. A., Heim H.-P., Feldmann M. and Akindoyo J. O., Advances in Polymer Technology, 2018

[cit143] Oliver-Ortega H., Llop M. F., Espinach F. X., Tarrés Q., Ardanuy M., Mutjé P. (2018). Composites, Part B.

[cit144] Venkatraman P., Gohn A. M., Rhoades A. M., Foster E. J. (2019). Composites, Part B.

[cit145] Annandarajah C., Langhorst A., Kiziltas A., Grewell D., Mielewski D., Montazami R. (2019). Materials.

[cit146] Beg M., Islam M. R., Mamun A., Heim H.-P., Feldmann M. (2019). Polym. Polym. Compos..

[cit147] Li Z., Shi T., Tan D. (2012). Polym.-Plast. Technol. Eng..

[cit148] Benaducci D., Branciforti M. C. (2016). Matéria.

[cit149] Peng S. X., Shrestha S., Youngblood J. P. (2017). Polymer.

